# Oxidized Lipids and Lysophosphatidylcholine Induce the Chemotaxis, Up-Regulate the Expression of CCR9 and CXCR4 and Abrogate the Release of IL-6 in Human Monocytes

**DOI:** 10.3390/toxins6092840

**Published:** 2014-09-23

**Authors:** Johannes Rolin, Heidi Vego, Azzam A. Maghazachi

**Affiliations:** Department of Physiology, Institute of Basic Medical Sciences, Faculty of Medicine, University of Oslo, Oslo 0317, Norway; E-Mail: heidi.vego@studmed.uio.no

**Keywords:** CCR9, CXCR4, HODE, LPC, monocytes, SDF-1α, TECK

## Abstract

Lipids through regulation of chronic inflammation play key roles in the development of various diseases. Here, we report that a mixed population of human primary monocytes migrated towards LPC, as well as oxidized linoleic acid isoforms 9-S-HODE, 9-R-HODE and 13-R-HODE. Incubation with 9-R-HODE, 13-R-HODE and LPC resulted in increased expression of CXCR4, the receptor for SDF-1α/CXCL12, correlated with increased monocyte migration towards SDF-1α/CXCL12. Further, we report increased expression of CCR9, the receptor for TECK/CCL25, after stimulation with these lipids. Upon examining the migratory response towards TECK/CCL25, it was observed that an increase in CCR9 expression upon pre-treatment with 9-S-HODE, 9-R-HODE, 13-R-HODE and LPC resulted in increased migration of monocytes expressing CCR9. Only LPC but not any other lipid examined increased the influx of intracellular Ca^2+^ in monocytes. Finally, 9-S-HODE, 9-R-HODE, 13-R-HODE, or LPC inhibited the release of IL-6 from monocytes suggesting that these lipids may play important role in controlling inflammatory responses.

## 1. Introduction

Lipids are important mediators of inflammation, contributing to diseases such as cancer and atherosclerosis [1,2,3]. For example sphingosine 1-phosphate (S1P), and polyunsaturated fatty acids, such as linoleic acid are abundant in the cell membranes, and in lipoproteins including low density lipoprotein “LDL” [4]. As a result of many different biological processes, they may be oxidized via enzymatic processes or lipid peroxidation [5,6]. Such processes result in the formation of new epitopes for recognition by immune cells, and a wide range of different pathways exist for immunological activation in response to lipids and their oxidation products [7,8,9,10]. 

Specific receptors such as the S1P receptors S1PR1-5 and LPA_1–4_ are examples of G-protein coupled receptors “GPCRs” which initiate intracellular signals leading to the activation of various cellular functions such as chemotaxis and cytokine production, among others [11,12]. On the other hand, scavenger receptors, such as CD36, identify numerous epitopes of oxidized lipids, as it was shown that 90% of the epitopes for this receptor were attributable to oxidized phospholipids, mainly oxidized phosphatidylcholine [13]. This is in line with several proposals suggesting that oxidized epitopes may represent danger associated molecular patterns “DAMPs” which are recognized by pattern recognition receptors “PRRs” present on innate immune cells [14,15]. Although products of many different enzymatic and non-enzymatic processes, most polyunsaturated fatty acid oxidation products yield identical oxidation products, regardless of the means of oxidation [16]. Accordingly, it was proposed that oxidation of lipids by acutely activated immune cells may be a controlled event with a central role in regulating innate immune functions during health and disease [17].

Recruitment and activation of innate immune cells, such as monocytes and neutrophils, by these lipids is highly important [18,19]. This is especially relevant in case of atherosclerosis, a chronic inflammatory disease in which the accumulation of monocytes, as well as oxidized lipids, is regarded as key pathogenic factors (reviewed in [20]). Because attraction of monocytes is a controlled event, several studies focused on understanding how oxidized lipids as compared to other inflammatory lipids take part in regulating the function of innate immune cells [21]. We recently examined the response of natural killer (NK) cells to lysophosphatidylcholine (LPC) and the linoleic acid oxidation products 9-S-HODE, 9-R-HODE and 13-R-HODE, and reported that these lipids were able to stimulate chemotaxis in these cells [22]. Based on the fact that monocytes and oxidized lipids co-localize in atherosclerotic plaques and due to observations of changes in monocyte function as well as indications of altered maturation when they were incubated with oxidized lipids, we sought to investigate whether the findings reported in NK cells may reflect wider distribution among cells of the innate immune system. In the current report, we investigated whether LPC and oxidized lipids may affect various activities of peripheral blood monocytes.

## 2. Results

### 2.1. Several Isoforms of HODEs and LPC Induce Chemotaxis of Primary Human Monocytes

**Figure 1:**
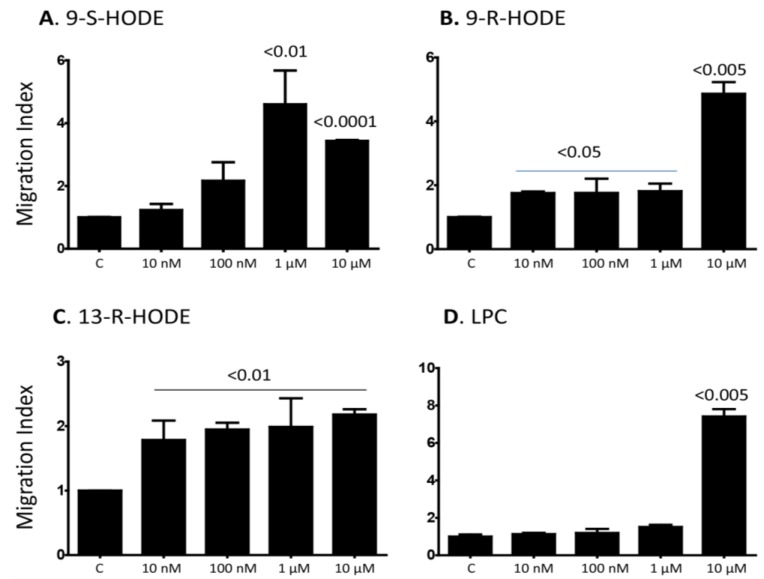
Various isoforms of HODE, and LPC induce the *in vitro* chemotaxis of human monocytes. (**A**) Various concentartions ranging between 0.01–10 µM of 9-S-HODE were placed in the lower wells of Boyden chmabers, wheraes 1 × 10^5^ monocytes were placed in the upper wells. Two hours later, the filters were collected, the cells fixed and then stained with modified Giemsa stain. Migration index (MI) was calculated as the numbers of cells migarting in the presence of the lipid divided by the numbers of cells migrating in the absence of the lipid (Control = C); (**B**) Similar to panel (**A**) except that 9-R-HODE was used; (**C**) Similar to panel (**A**) except that 13-R-HODE was used; (**D**) Similar to panel (**A**) except that LPC was used. Mean ± SEM of 5 experiments performed. *p* values comparing the effect of the lipids *vs.* the control are shown on top of the columns.

To demonstrate that primary human monocytes are affected by the lipids, we first confirmed that these cells contained about 90% CD14^+^, less than 5% CD3^+^ T cells and less than 1% CD19^+^ B cells as determined by flow cytometric analysis (Figure S1). Next, we examined whether oxidized lipids and LPC induce the *in vitro* monocyte chemotaxis. Our results show that 1 and 10 µM of 9-S-HODE induced chemotaxis (*p* < 0.01 and <0.0001, respectively as compared to the control, Figure 1A). In addition, 0.01–10 µM of 9-R-HODE and 13-R-HODE induced their chemotaxis (Figure 1B,C, respectively). On the other hand, only the highest concentration, *i.e.*, 10 µM of LPC induced monocyte chemotaxis (*p* < 0.005, Figure 1D). These results indicate that several HODEs as well as LPC induce the chemotaxis in monocytes although at different concentrations, suggesting that the lipids might have different affinities for the receptor, or they may utilize different receptors. 

### 2.2. LPC Induces the Mobilization of Intracellular Calcium in Primary Human Monocytes

**Figure 2:**
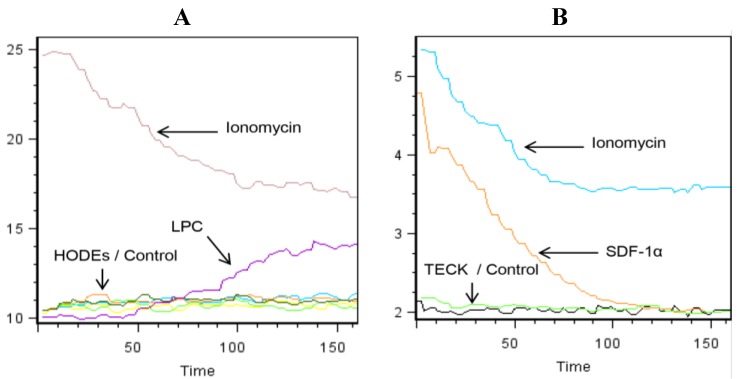
LPC and CXCL12/SDF-1α induce the mobilization of intracellular calcium in human monocytes. Freshly isolated monocytes were rested overnight, harvested and kept on ice. Immediately prior to running, samples were re-suspended in a pre-heated buffer containing RPMI plus 0.1% BSA and 1mM CaCl_2_ and rested for one minute. Then 50 µM of 9-S-HODE, 9-R-HODE, 13-R-HODE or LPC (**A**); or 100 ng/mL of TECK/CCL25 or SDF-1α/CXL12 (**B**) was added, and the samples examined for 120 s in a flow cytometer. Representative of 3 experiments performed. Black oscillations indicate control (media only), whereas other colors in panel (**A**) show the effect of various HODEs, and green oscillations in panel (**B**) show the effect of TECK/CCL25.

Next, we examined whether the lipids that augment chemotaxis of monocytes may also induce the mobilization of intracellular Ca^2+^ in these cells. For control, Ionomycin and two chemokines, namely TECK/CCL25 and SDF-1α/CXCL12 were used. Monocytes were rested overnight, labeled at 1 × 10^6^ cells/mL for 45 min at 37 °C with 0.8 µM Indo-3 AM, washed, and kept on ice. Before stimulation, the cells were resuspended at 1 × 10^6^ cells/mL in a buffer containing 1 mM CaCl_2_. They were rested for 1 min at 37 °C, stimulated with various concentrations of the lipids or chemokines and immediately examined in the flow cytometer for 120 s. Results show that Ionomycin induced a robust mobilization of calcium (Figure 2, panels A,B). 9-S-HODE, 9-R-HODE, 13-R-HODE and LPC were used at several concentrations. Among the lipids examined, only LPC induced the mobilization of intracellular calcium (Figure 2A). On the other hand, SDF-1α/CXCL12 but not TECK/CCL25 induced the mobilization of intracellular calcium in these cells (Figure 2B).

### 2.3. Oxidized Lipids and LPC Increase the Expression of CCR9 and CXCR4 on the Surface of Monocytes

Due to observations suggesting a regulatory role of oxidized lipids as well as LPC on chemokine receptor expression in immune cells, we sought to examine the effects of these lipids on the expression of chemokine receptors in monocytes. Consequently, human primary monocytes were incubated with 20 µM concentration of 9-S-HODE, 9-R-HODE, 13-R-HODE, or LPC for 4 and 24 h, or with media as a control.

**Figure 3:**
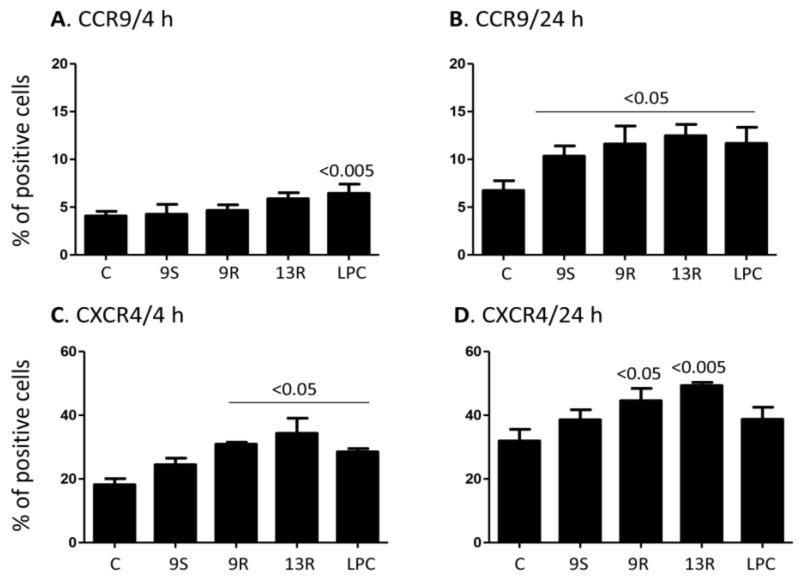
Lipids up-regulate the expression of CCR9 and CXCR4 on the surface of monocytes. (**A**) Monocytes were treated for 4 h with 20 µM of 9-S-HODE, 9-R-HODE, 13-R-HODE and LPC or with media only (Control = C). The cells were washed and then examined for the expression of CCR9; (**B**) Similar to panel (**A**) except that the cells were incubated with the lipids for 24 h; (**C**) Monocytes were treated for 4 h with 20 µM of 9-S-HODE, 9-R-HODE, 13-R-HODE, and LPC or with media only (Control = C). The cells were washed and then examined for the expression of CXCR4; (**D**) Similar to panel (**C**) except that the cells were incubated with the lipids for 24 h. Mean ± SEM of 5 experiments performed. *p* values comparing the effect of lipids *vs.* the control are shown on top of the columns.

**Figure 4:**
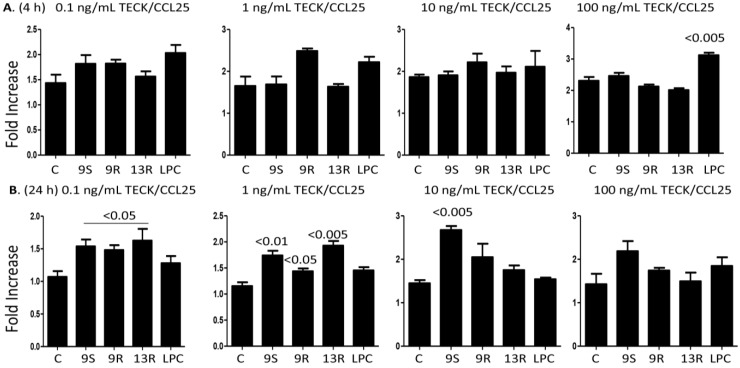
Monocytes pre-treated with the lipids migrate towards the concentration gradients of TECK/CCL25. (**A**) Monocytes were incubated for 4 h with 20 µM of 9-S-HODE, 9-R-HODE, 13-R-HODE, LPC or with media only. The cells were washed and then incubated in the upper wells of Boyden chambers. In the lower wells 0.1, 1, 10 or 100 ng/mL of TECK/CCL25 was placed; (**B**) Similar to the upper panels except that the cells were pre-treated with the lipids for 24 h. Filters were collected, stained and the numbers of the cells counted. Migration index (MI) was calculated as the number of cells migrating in the presence of the chemokine divided by the number of cells migrating in its absence. Fold increase indicates the increase of MI towards the chemokine after pre-treatment with the lipids *vs.* the MI obtained towards the chemokine in the absence of lipids pre-treatment (indicated as control = C). Mean ± SEM of 5 experiments performed. *p* values comparing the effect of lipids *vs.* the control are shown on top of the columns.

Of all the chemokine receptors examined which include CCR1, CCR2, CCR3, CCR4, CCR5, CCR6, CCR7, CCR8, CCR9, CCR10, CXCR1, CXCR2, CXCR3, CXCR4, CXCR5, CXRC6, and CX_3_CR1, we observed effects on CCR9 and CXCR4 expression only. Our results show that incubation of monocytes with 20 µM of LPC, but not any other lipid, for 4 h significantly induced increased expression of CCR9 (*p* < 0.005, Figure 3A). However, incubation with 20 µM for 24 h of 9-R-HODE, 9-S-HODE, 13-R-HODE or LPC increased the expression of CCR9 relative to the expression in cells incubated with media only (*p* < 0.05 for all lipids, Figure 3B). The level of CXCR4 expression was also increased after 4 h when cells were treated with 20 µM of 9-R-HODE, 13-R-HODE or LPC (*p* < 0.05, Figure 3C). Further, incubation for 24 h with 20 µM of 9-R-HODE or 13-R-HODE also significantly increased the expression of CXCR4 at this time point (Figure 3D). Of note, 9-S-HODE was without effect and the increased expression observed with LPC after 4 h was lost after 24 h incubation (Figure 4D).

### 2.4. Oxidized Lipids and LPC Augment Monocyte Chemotaxis towards TECK/CCL25

In order to assess the functional relevance of the increase in the expression of CCR9, we performed chemotaxis experiments towards TECK/CCL25. Because monocytes untreated with the lipids also migrated towards the concentrations gradients of the chemokines, we present the results as fold increase of chemotaxis towards various concentrations of TECK/CCL25 in cells pre-treated with 20 µM of the lipids as compared to migration in the absence of pre-treatment with the lipids. Results in Figure 4A indicate that cells pre-treated with 20 µM of LPC significantly increased migration towards the 100 ng/mL concentration of TECK/CCL25 when compared to cells migrating towards the same concentration of the chemokine but without pre-treatment with any of the lipids (C = control). These results corroborate with the ability of LPC to significantly increase the expression of CCR9 on the surface of monocytes 4 h after incubation.

Pre-treatment for 24 h with 9-S-HODE, 13-R-HODE and 9-R-HODE also increased monocyte migration towards 0.1 and 1 ng/mL concentrations of TECK/CCL25 (Figure 4B), in line with the ability of these lipids to increase the expression of CCR9 on the surface of these cells after 24 h incubation (see Figure 3B). Unexpectedly, only 9-S-HODE significantly increased their chemotaxis towards 10 ng/mL of the chemokine, an activity that disappeared when 100 ng/mL of the chemokine was used (Figure 4B). Perhaps the 100 ng/mL of this chemokine may induce the desensitization of the receptor but this only occurs after 24 h incubation, suggesting that CCR9 might adapt a higher affinity towards its ligand TECK/CCL25 after overnight incubation with the lipids.

### 2.5. Oxidized Lipids and LPC Induce Increased Chemotaxis towards SDF-1α/CXCL12

**Figure 5:**
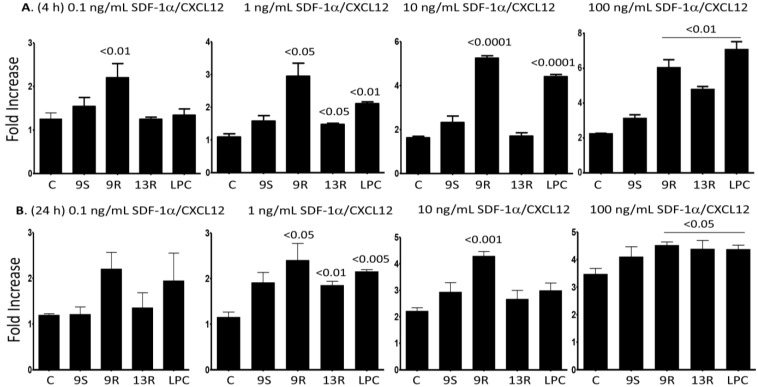
Monocytes pre-treated with the lipids migrate towards the concentrtion gradients of SDF-1α/CXCL12. (**A**) Monocytes were incubated for 4 h with 20 µM of 9-S-HODE, 9-R-HODE, 13-R-HODE, LPC or media only. The cells were washed and then incubated in the upper wells of Boyden chambers. In the lower wells 0.1, 1, 10 or 100 ng/mL of SDF-1α/CXCL12 was placed; (**B**) Similar to the panels shown in (**A**), except that the cells were pre-treated with the lipids for 24 h. Filters were collected, stained and the cells counted. Migration index (MI) was calculated as the numbers of cells migarting in the presence of the chemokine divided by the numbers of cells migrating in the absence of chemokine. Fold increase indicates the increase of MI towards the chemokine after pre-treatment with the lipids *vs.* the MI obtained towards the chemokine in the absence of lipids pre-treatment (indicated as control = C). Mean ± SEM of 5 experiments performed. *p* values comparing the effect of lipids *versus* the controls are shown on top of the columns.

In order to assess the functional relevance of the observed up-regulation of CXCR4 by the lipids, we performed chemotaxis experiments. After 4 h pre-treatment with the lipids, increased chemotaxis towards 1, 10, and 100 ng/mL of SDF-1α/CXCL12 was observed, when compared to the chemotaxis of cells towards the same concentration of the chemokine but without lipids pre-treatment; an exception is the effect of 13-R-HODE on the migration towards the 10 ng/mL of the chemokine (Figure 5A). In accordance with increased expression of CXCR4, pre-treatment of monocytes with 9-R-HODE, 13-R-HODE or LPC for 24 h also increased their migration towards 1, 10 and 100 ng/mL of the ligand for CXCR4, SDF-1α/CXCL12 (Figure 5B). Of note, we did not observe an increase in monocyte chemotaxis when these cells were pre-treated with 9-S-HODE for 4 h or 24 h, corroborated with the inability of this lipid to up-regulate the expression of CXCR4 on the surface of the cells (see Figure 3).

### 2.6. Oxidized Lipids and LPC Inhibit IL-6 Release from Monocytes

**Figure 6:**
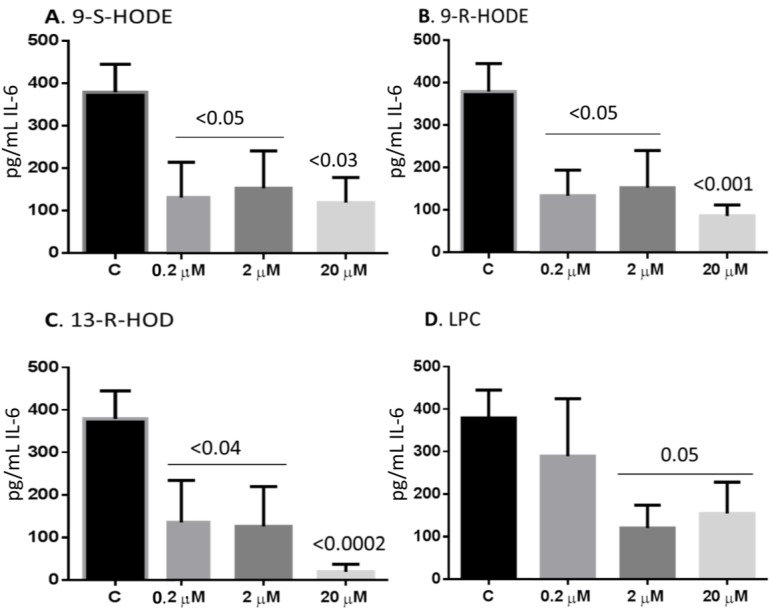
Oxidized lipids and LPC inhibit IL-6 secretion from monocytes. Monocytes were incubated at a cell concentration of 1 × 10^6^ cells/mL with media or with 200 nM, 2 µM or 20 µM of 9-S-HODE (**A**); 9-R-HODE (**B**); 13-R-HODE (**C**); or LPC (**D**). After 24 h incubation, the cells were harvested and the cell suspensions were centrifuged and the supernatants were collected. Levels of IL-6 were determined according to the standards provided by the manufacturer. Mean ± SEM of 3 experiments.

Finally, we sought to examine the impact of the lipids on the secretion of cytokines. Preliminary ELISAarray analysis indicates that the lipids exerted no effect on the levels of inflammatory cytokines and chemokines IL-1β, IL-4, IL-10, IL-12, IFN-γ, TNF-α, CCL2, CCL3 and CCL4, but affected the release of the pro-inflammatory cytokine IL-6 (Figure S2). Consequently, we examined in details the effects of various concentrations of the lipids on the release of IL-6 by monocytes. Supernatants were collected 24 h after incubating monocytes with media or with the lipids and analyzed for the levels of IL-6. Untreated monocytes robustly secreted IL-6, an effect that was significantly reduced by pre-treatment with all lipids. Cells pre-treated with 0.2–2 µM of 9-S-HODE reduced the secretion of IL-6 to less than half (Figure 6A). Cells pre-treated with all three concentrations of 9-R-HODE showed a significant reduction in the release of IL-6 (Figure 6B). On the other hand, pre-treatment with 20 µM of 13-R-HODE completely abrogated the secretion of IL-6, while the lower concentrations of this lipid significantly inhibited its secretion (Figure 6C). Incubation with 2 and 20 µM of LPC also significantly inhibited IL-6 release (Figure 6D)

## 3. Discussion

In this communication, we report that oxidized lipids including 9-S-HODE, 9-R-HODE and 13-R-HODE, as well as LPC, induce the *in vitro* chemotaxis of monocytes, similar to what we described earlier regarding the effects of these lipids on the chemotaxis of NK cells [22]. This effect was observed with rather higher concentrations of the lipid, for example 20 µM. However, this is not surprising since others reported activities with similar or even higher concentrations. Nagy *et al.* [23] reported a dose-dependent activation of peroxisome proliferator-activated receptor-γ “PPAR-γ” in human monocytes in the range of 2.5–60 µM oxLDL. They suggested a Kd for 9-HODE and 13-HODE in the range of 10–20 µM. The authors further observed an increase in the expression of CD14 and CD36 molecules over four days of stimulation with 15 µM 9–HODE or 13-HODE. Huang *et al.* [24] obtained similar results by exposing macrophages to 20 or 50 µM of 13-HODE, whereas others observed activation of human trophoblasts in a culture with 20 µM 9–HODE or 13-HODE [25]. On the other hand, it was shown that 9-HODE activates the G-protein coupled receptor GPCR132 (G2A, G2 accumulation) with a half- maximum effect at the low concentration of 2 µM, and a maximum effect at 10 µM [26]. Concentrations of these lipids *in vivo* are largely considered unknown, but some attempts have been made to quantify them. The total content of HODE in tissues was estimated at about 51 ng/g in plaques, which gives a molecular weight of 297 corresponding to a concentration of about 40–170 µM [27,28].

There is uncertainty about the nature of the receptors binding these lipids. In case of LPC, a controversy whether this lipid might bind G2 accumulation (G2A) was reported [29]. However, it was also reported that G2A expression was necessary for the migration of macrophages towards LPC [8], and neutrophils expressing this receptor respond with influxing calcium upon stimulation with LPC [30]. Further, we previously reported partial desensitization between LPC and 9-S-HODE or 9-R-HODE [22]. Regarding the effects on the mobilization of intracellular calcium in NK cells, abrogation of the effects of these lipids by pertussis toxin was observed, suggesting that the action of these lipids may involve a GPCR. Here, we observed that LPC behaves differently than oxidized lipids: (1) LPC, but not 9-S-HODE, 9-R-HODE, or 13-R-HODE, mobilizes intracellular calcium in primary human monocytes; and (2) Only LPC up-regulates the expression of CCR9 on the surface of monocytes after 4 h stimulation, resulting in their enhanced chemotaxis towards TECK/CCL25 at this time point. These findings suggest that in monocytes LPC may bind different receptor(s) than oxidized lipids, or that the receptor(s) may couple to different G proteins. Calcium and chemotaxis are different processes; for example calcium influx is a fast process that takes few seconds to complete and it requires different G proteins than those mediating cell chemotaxis which takes a longer time to start [31]. Further, 9-S-HODE did not up-regulate the expression of CXCR4 on monocytes, and neither promoted their chemotaxis towards SDF-1α/CXCL12. Collectively, these results emphasize the differential effects exerted by these lipids on monocytes.

Oxidized lipids decrease CCR2 expression [32], and increase CX_3_CR1 expression in monocytes [33], while they induce increased CCR7 expression in immature dendritic cells [34], emphasizing the immune-modulatory role of these lipids. Here, we observed an increase in the expression of CXCR4 in primary monocytes after pre-treatment with 9-R-HODE, 13-R-HODE and LPC for 4 h, an effect that is even stronger after 24 h incubation. Further, these lipids induced directed migration of monocytes towards SDF-1α/CXCL12 after similar time of pre-treatment with the lipids. Our observations are in line with the observations of others who showed increased CXCR4 expression in human CD4^+^ T cells [35]. However, such effect has not been previously shown in monocytes. 

Expression of SDF-1α/CXCL12 is increased in experimental atherosclerosis [36], and expression of SDF-1α/CXCL12 following arterial injury is an important early step in the development of atherosclerosis [37]. As the disease progresses, this chemokine is expressed at high levels in smooth muscle cells, endothelial cells as well as macrophages in atherosclerotic plaques, but it is not present in normal vessels [38]. Emphasizing its relevance through the course of disease progression, SDF-1α/CXCL12 was the only chemokine attracting platelets and induced their aggregation [38]. The importance of CXCR4 has also been studied in the context of cancer [39]. In pancreatic cancer, a subset of cells expressing CXCR4 was identified as an important determining factor for the metastatic phenotype of individual tumors [40]. The mechanism by which hematopoietic stem cells use CXCR4 to reach bone marrow niches, is exploited by CXCR4 positive leukemic cells to obtain access to a favorable growth environment [39]. In monocytes, an increase in CXCR4 expression has been shown in CD14^+^CD16^−^ cells cultured with cancer cells, alongside increases in the expression of CCR2, CXCR1 and CXCR2 [41]. Additionally, when monocytes were grown in a hypoxic environment, the CXCR4 expression was up-regulated [42].

Ovarian cancer cells secrete SDF-1α/CXCL12, which led to the suggestion that through such mechanisms plasmacytoid dendritic cell precursors are recruited resulting in poor T cell activation [43]. In line with these observations, our results showing that oxidized lipids and LPC induce the chemotaxis of these cells and up-regulate the expression of CXCR4 may reflect development towards a population of monocytes that have important implications for atherosclerosis and cancer development.

In addition, we observed increased expression and function of CCR9, the receptor for TECK/CCL25. CCR9-expressing monocytes comprise a pro-inflammatory subset in inflammatory bowel disease [44], and also increased in arthritis patients [45]. The percentages of CCR9 positive cells in inflamed synovial fluid were more than doubled as compared to the blood of arthritis patients. Interestingly, in the controls there were more than four times as many CCR9^+^ cells in the synovial fluid, suggesting that these cells are preferentially recruited due to factors secreted at these sites, and also due to up-regulation of CCR9 once the cells migrated into inflammatory sites [46]. An investigation of the phosphatidylcholine/lysophosphatidylcholine ratio in arthritic patients showed increased levels of LPC in blood as well as in synovial fluids and the level of LPC determined the severity of joint disease [47]. This could reflect the general hyperlipidemia of atherosclerotic patients and may explain the increase in CCR9 expression in peripheral blood of arthritis patients. Indeed, LPC is elevated in a number of different diseases [48], but in the blood of septic patients, LPC production was increased, corroborated with increased survival [49]. Because sepsis is an acute inflammation which is different from chronic diseases, an increase in LPC may reflect attempts of the immune system to combat inflammation, which is detrimental in cases of chronic, self-perpetuating inflammation such as arthritis and atherosclerosis. Thus, migration towards LPC may reflect a mean of recruiting monocytes into inflammatory sites in order to mount a protective response. Our observation showing up-regulation of CCR9 expression in monocytes treated with oxidized lipids and LPC and their directed chemotaxis towards these lipids might provide some explanations to these findings.

Although it was earlier reported that LPC but no other lipids stimulates IL-6 release from rat anterior pituitary cells [50], other findings shed more light on the role of this lipid and oxidized lipids in monocytes/macrophages. For example, Jiang *et al.*, observed that agonists of PPAR-γinhibited the production of TNF-α, IL-1β and IL-6 from monocytes [51]. Our findings showing that LPC or HODEs inhibit the release of IL-6 from human monocytes are in line with these observations. It was previously shown that LPC promoted cellular cholesterol efflux from human macrophages by activating PPAR-γ [52]. Similarly 9-R-HODE and 13-R-HODEs are natural ligands for PPAR-γ [53]. Hence, LPC and HODEs inhibit the release of IL-6 by monocytes perhaps by activating PPAR-γ in these cells, although this was not examined. However, these findings add to the concept that lipids may exert protective effects at sites of injury. We previously reported that other lysophospholipids, such as LPA and S1P, induce the release of IL-6 from maturing but not mature DCs [54], results that should not contradict the present findings since the lipids and the cell types used are different among the two studies.

In summary, we observed that LPC and oxidized lipids promote the chemotaxis of monocytes and up-regulate the expression of CCR9 and CXCR4 corroborated with enhanced chemotaxis of these cells towards the ligands for these chemokines, *i.e.*, TECK/CCL25 and SDF-1α/CXCL12, respectively. We propose that at inflammatory sites which include atherosclerotic plaques or tumor growth sites, these lipids might exert anti-inflammatory effects such as inhibiting the release of the pro-inflammatory cytokine IL-6 by recruited monocytes.

## 4. Experimental Section

### 4.1. Reagents

9-S-HODE, 9-R-HODE, 13R-HODE, and LPC were obtained from Cayman Chemicals (Ann Arbor, MI, USA). FITC-conjugated mouse anti-human CCR3, CCR4, CCR5, CCR6, CCR7, CCR9, CXCR1, CXCR3, CXCR4, and CXCR5 or unconjugated monoclonal mouse-anti-human CCR1, CCR2, and CXCR6, as well as PE-conjugated rat anti-human CCR8 and PE-conjugated rat IgG2b , were obtained from R&D Systems Europe Ltd (Abingdon, UK). FITC-conjugated mouse anti-human CX_3_CR1 was purchased from Medical and Biological Laboratories Co. Ltd (Nagoya, Japan). Unconjugated mouse anti-human HLA-class I, HLA-E or IgG1 as a control were obtained from eBioscience (San Diego, CA, USA). FITC-conjugated goat anti mouse was purchased from Beckton-Dickinson (San Diego, CA, USA) and FITC-conjugated mouse anti-human CD14 from Immunotools (Friesoythe, Germany). FITC-conjugated mouse IgG1, unconjugated mouse IgG1 and unconjugated rat IgG were obtained from either Becton-Dickinson or from R & D Systems.

### 4.2. Preparation and Culture of Cells

Monocytes were prepared as earlier described [55]. Briefly, peripheral blood cells were collected from blood bank healthy volunteers (Ullevål Hospital, Oslo, Norway) and centrifuged over Histopaque gradients (Sigma Aldrich, Oslo, Norway). Mononuclear cells were isolated and incubated at 1 × 10^7^/mL in 100-mm Petri dishes with total volume 10 mL or 60-mm Petri dishes with total volume 3 mL at 37 °C for 2 h, and the adherent cells were collected and examined. Freshly isolated monocytes were left intact or incubated with various concentrations of 9-S-HODE, 9-R-HODE, 13-R-HODE or LPC for 4 h or 24 h. The cells were extensively washed and then examined for various activities.

### 4.3. In Vitro Chemotaxis Assay

Nucleopore blind well chemotaxis chambers with a lower well volume of 200 μL were used. A maximum volume of 200 μL medium containing RPMI 0.1% BSA was placed in the lower wells in the presence or absence of various chemokines or lipids. Cells (2 × 10^5^) were placed in the upper compartments and incubated for 2 h at 37 °C in a 5% CO_2_ incubator. The filters (Nucleopore Polycarbonate 13 mm size 8 UM, Whatman International Ltd., Kent, UK), were removed, dehydrated, stained with 15% modified Giemsa stain for 7 min, and then mounted on glass slides. Cells in three high power fields were counted and migration index (MI) was calculated as the number of cells migrating towards the concentration gradients of chemokines divided by the number of cells migrating towards medium only as previously described [56].

### 4.4. Flow Cytometric Analysis

Freshly isolated monocytes were left intact or incubated with various concentrations of 9-S-HODE, 9-R-HODE, 13-R-HODE or LPC for 4 h or 24 h. The cells were washed and incubated in a 96-well plate (v-bottom, 2 × 10^5^ cells per well), washed again and resuspended in PBS buffer containing 0.1% sodium azide. Cells were labeled with antibodies at optimal concentrations, washed twice, and examined in the flow cytometer (FACSCalibur, Becton-Dickinson Biosciences, San Jose, CA, USA). Markers were set according to the isotype control FITC- or PE-conjugated mouse IgG.

### 4.5. Mobilization of Intracellular Calcium

Freshly isolated monocytes were rested overnight, harvested and incubated at a concentration of 10 × 10^6^ cells/mL with 0.8 µM of Fluo-3 AM for 45 min in a medium containing RPMI plus 0.1% BSA at 37 °C. The cells were distributed in samples of 3 × 10^5^ cells, pelleted and incubated on ice. They were resuspended in a preheated buffer containing RPMI plus 0.1% BSA and 1 mM CaCl_2_, and rested for one min at 37 °C. The different stimuli were added immediately before examining in the flow cytometer (FACSCalibur, Becton-Dickinson Biosciences, San Jose, CA, USA). As a positive control, 1.4 µM Ionomycin (Sigma-Aldrich, Oslo, Norway) was used.

### 4.6. Detection of Cytokines and Chemokines Release Utilizing the ELISArray Kits

Monocytes were incubated at a cell concentration of 1 × 10^6^ cells/mL with media or with 20 µM of the various lipids for 24 h. The cells were harvested and the cell suspensions were centrifuged for 10 min before the supernatants were collected. Detection of the levels of various cytokines and chemokines was carried utilizing the Multi Analyte ELISArray Kit (SA Biosciences, Frederick, MD, USA) as described by the manufacturers’ user manual. The kit analyzes the release of IL-1β, IL-4, IL-6, IL-10, IL-12, IFN-γ, TNF-α, MCP-1/CCL2, MIP-1α/CCL3, and MIP-1β/CCL4.

### 4.7. Detection of IL-6 Release by ELISA

Monocytes (1 × 10^6^ cells/mL) were incubated with media or with various concentrations of 9-S-HODE, 9-R-HODE, 13-R-HODE, or LPC for 24 h. The cells were harvested and the cell suspensions were centrifuged at 1000× *g* for 12 min before the supernatants were collected. Detection of the levels of various cytokines and chemokines was carried out utilizing the IL-6 ELISA kit (Antibodies-online GmbH, Aachen, Germany) as described by the manufacturers’ user manual. Controls supplied by the kit were also used.

### 4.8. Statistical Analysis

For Figure 1, Figure 2, Figure 3, Figure 4 and Figure 5, significant values were generated using Student’s *t*-test calculated by Graphpad Prism Program (Version 6, San Diego, CA, USA, 2014). For Figure 6, comparison was made among the control and treatment groups. For this, the one-way ANOVA corrected for multiple comparisons using Dunnell’s test was utilized.

## 5. Conclusions

This is the first report showing that LPC and oxidized lipids up-regulate certain chemokine receptors, in particular CCR9 or CXCR4 on the surface of monocytes, and facilitate their chemotaxis towards TECK/CCL25 of SDF-1α/CXCL12. In addition, these lipids can *per se* recruit monocytes. These combined effects are so potent allowing monocytes to accumulate at sites of inflammation, especially in diseases, such as atherosclerosis and cancer. Further, these lipids inhibit the release of IL-6 from these same monocytes. Such effects should encourage performing more experiments in order to dissect the activities of lipids in more details for the purpose of tipping the balance towards a beneficial outcome.

## Supplementary Materials

Supplementary materials can be accessed at: http://www.mdpi.com/2072-6651/6/9/2840/s1.

## Supplementary Files

Supplementary File 1
