# Supplementary material for: Oxidized Lipids and Lysophosphatidylcholine Induce the Chemotaxis, Up-Regulate the Expression of CCR9 and CXCR4 and Abrogate the Release of IL-6 in Human Monocytes
Source: Toxins (Basel). 2014 Sep 23;6(9):2840–56. doi: 10.3390/toxins6092840 (PMC4179163; doi:10.3390/toxins6092840)

Supplementary Information

Figure S1. Phenotype of isolated adherent cells.

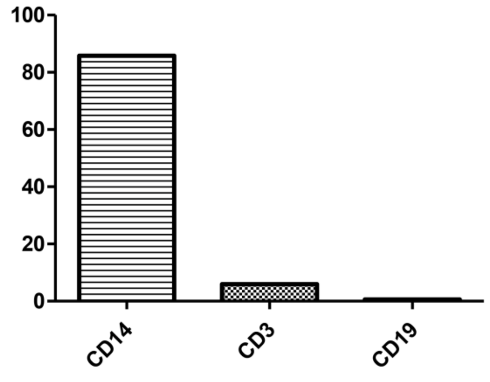

Figure S2. Effect of the lipids on the release of inflammatory cytokines and chemokines by monocytes. Monocytes were incubated with 20 µM of the lipids for 24 h. Supernatants were collected and examined for the release of various cytokines and chemokines.

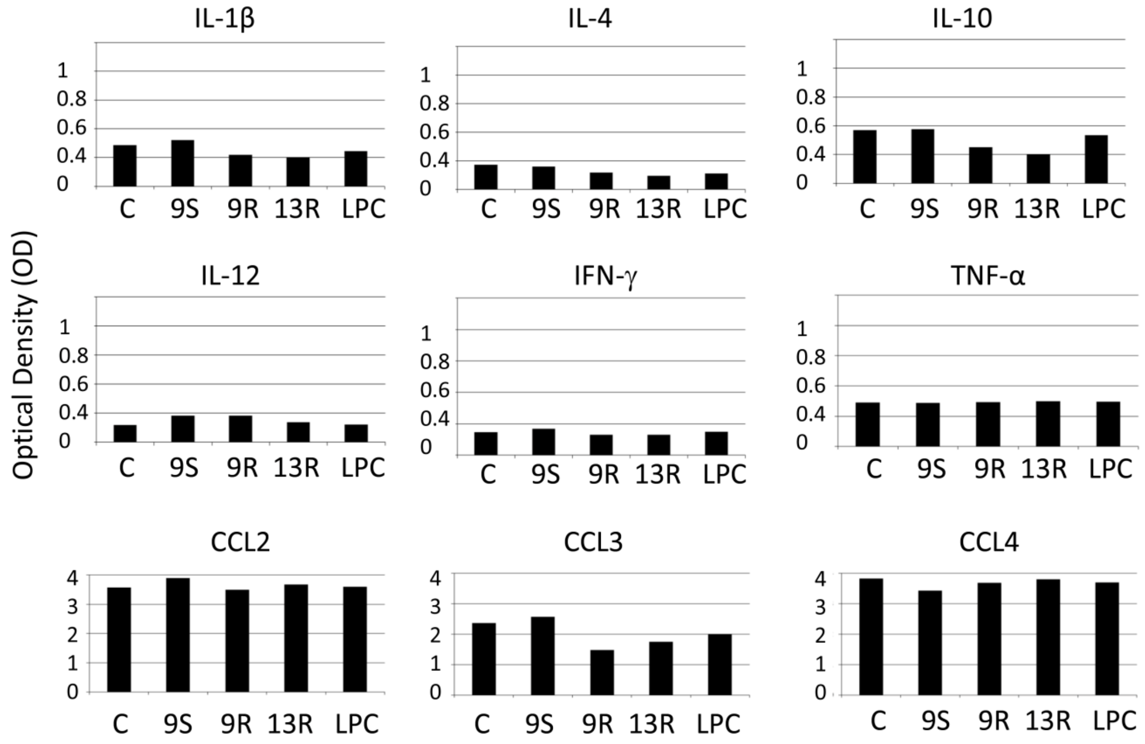

Supplement: Supplementary File 1 [file toxins-06-02840-s001.pdf]
